# Engineering Gain-of-Function Analogues of the Spider Venom Peptide HNTX-I, A Potent Blocker of the hNa_V_1.7 Sodium Channel

**DOI:** 10.3390/toxins10090358

**Published:** 2018-09-04

**Authors:** Yunxiao Zhang, Qiuchu Yang, Qingfeng Zhang, Dezheng Peng, Minzhi Chen, Songping Liang, Xi Zhou, Zhonghua Liu

**Affiliations:** The National & Local Joint Engineering Laboratory of Animal Peptide Drug Development, College of Life Sciences, Hunan Normal University, Changsha 410081, Hunan, China; Zhangyx0954@163.com (Y.Z.); QQQQiuchu@126.com (Q.Y.); feng122436ab@126.com (Q.Z.); pdz920217@sina.com (D.P.); chenmz@hunnu.edu.cn (M.C.); liangsp@hunnu.edu.cn (S.L.)

**Keywords:** voltage-gated sodium channels, Na_V_1.7, spider venom, toxin, HNTX-I, engineering

## Abstract

Pain is a medical condition that interferes with normal human life and work and reduces human well-being worldwide. Human voltage-gated sodium channel NaV1.7 (hNaV1.7) is a compelling target that plays a key role in human pain signaling. The 33-residue peptide µ-TRTX-Hhn2b (HNTX-I), a member of Na_V_-targeting spider toxin (NaSpTx) family 1, has shown negligible activity on mammalian voltage-gated sodium channels (VGSCs), including the hNa_V_1.7 channel. We engineered analogues of HNTX-I based on sequence conservation in NaSpTx family 1. Substitution of Asn for Ser at position 23 or Asp for His at position 26 conferred potent activity against hNa_V_1.7. Moreover, multiple site mutations combined together afforded improvements in potency. Ultimately, we generated an analogue E1G–N23S–D26H–L32W with >300-fold improved potency compared with wild-type HNTX-I on hNa_V_1.7 (IC_50_ 0.036 ± 0.007 µM). Structural simulation suggested that the charged surface and the hydrophobic surface of the modified peptide are responsible for binding affinity to the hNa_V_1.7 channel, while variable residues may determine pharmacological specificity. Therefore, this study provides a profile for drug design targeting the hNa_V_1.7 channel.

## 1. Introduction

Pain treatment causes significant health concerns and economic burdens for affected families and for society [1,2,3]. Currently available drugs have safety and tolerability limitations and are often ineffective or suboptimally used [4,5,6]. There is therefore a critical unmet medical need to discover novel potential analgesia candidates, as well as to improve currently available therapies for severe and chronic pain [7].

Human Na_V_1.7 (hNa_V_1.7) has been identified as an important channel playing a crucial role in human pain signaling [8]. It is preferentially expressed in peripheral neurons, including peripheral somatic and visceral sensory neurons within the dorsal-root ganglia, sympathetic ganglion neurons, myenteric neurons and olfactory–sensory neurons [9,10]. In humans, gain-of-function mutations of hNa_V_1.7 lead to severe neuropathic pain, such as inherited erythromelalgia (IEM) [11,12,13], paroxysmal extreme pain disorder (PEPD) [14,15] and idiopathic small-fiber neuropathy (SFN) [16], while loss-of-function mutations cause congenital insensitivity to pain (CIP) [17]. Based on the results of validated and compelling genetic studies, hNa_V_1.7 has become an outstanding target for developing new therapies for pain [18].

Natural peptide toxins from animal venom have been an invaluable and original source of novel, selective and potent modulators targeting ion channels implicated in pain signaling pathways [19]. Indeed, some peptide toxins derived from animal venoms inhibit the hNa_V_1.7 channel and exhibit remarkable analgesia. Spider venom–derived peptide toxins can be divided into 12 distinct families that target Na_V_ channels based on primary structure and cysteine scaffold. Peptides in Na_V_-targeting spider toxin (NaSpTx) family 1 contain 33–35 amino acid residues, with 3 disulfide bridges forming an inhibitory cystine-knot (ICK) motif., Through the alignment and structural analysis of conserved sequences, it is possible to rationally design novel peptides that are likely to inhibit the Na_V_1.7 channel.

Previously, we reported the identification of 33-residue peptide µ-TRTX-Hhn2b (HNTX-I), a member of NaSpTx family 1, from the venom of the spider *Selenocosmia hainana*. Although the amino acid sequence of this family is highly conserved, HNTX-I has shown weak affinity on insect sodium channels and no effect on mammalian voltage-gated sodium channels (VGSCs), unlike the majority of peptide toxins in NaSpTx family 1 [20]. In this study, we used HNTX-I as a framework and engineered analogues of it based on sequence conservation in the family. Ultimately, we engineered a gain-of-function analogue of HNTX-I: E1G-N23S-D26H-L32W, a potent blocker of the hNa_V_1.7 channel with an IC_50_ value of 0.036 ± 0.007 µM. Our data define the motif X_1_X_2_SWCKX_3_ as critical for suppressing the hNa_V_1.7 channel.

## 2. Results

### 2.1. Sequence Alignment of HNTX-I in NaSpTx Family 1

**Figure 1:**
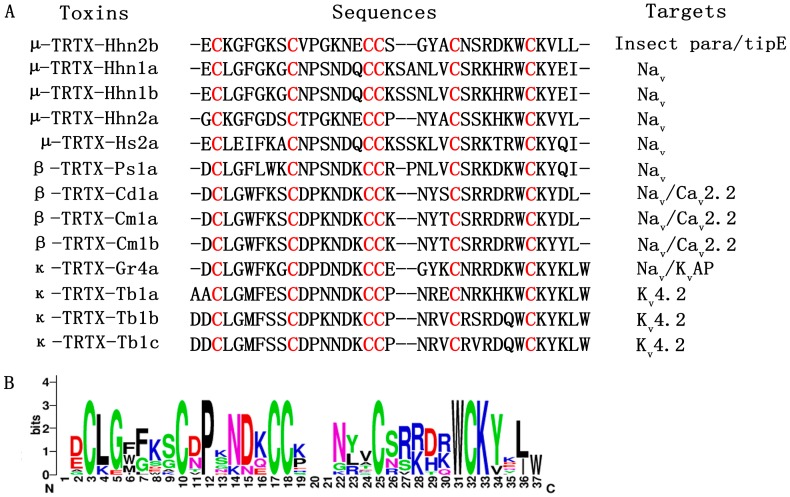
Sequence alignment of peptide toxins in the NaSpTx family 1. (**A**) Alignment of mature toxin sequences in NaSpTx family 1. Cysteines are highlighted in red; (**B**) Sequence logo for alignment in NaSpTx family 1. Residues shown in black are hydrophobic, those shown in red are negatively charged, those shown in blue are positively charged, and those shown in green are polar uncharged. The overall height of the stack indicates the sequence conservation at that position, while the height of letter within the stack indicates the relative frequency of each amino acid.

Figure 1A shows the amino acid sequence alignment of peptides in NaSpTx family 1. Conserved cystine residues are highlighted in red. These peptides form an ICK motif that is typically resistant to extremes of pH, organic solvents, high temperatures and proteases [21]. A sequence logo for this alignment is displayed in Figure 1B. The overall height of the stack indicates sequence conservation at that position, while the height of the letter within the stack indicates the relative frequency of each amino acid at that position. Proline at position 12, tryptophan at position 31 and lysine at position 33 are not buried, indicating that residues here appear to be critical for peptide activity. Positions that are partially or completely buried tend to be populated by the corresponding residues. For example, serine is more popular in position 26, as is aspartic acid in position 29. In particular, the serine in position 26 has been demonstrated to be important for activity [22,23], which validates the feasibility of modifying peptide activity according to sequence conservation.

### 2.2. Rational Design of HNTX-I in Consideration of Sequence Conservation

**Figure 2:**
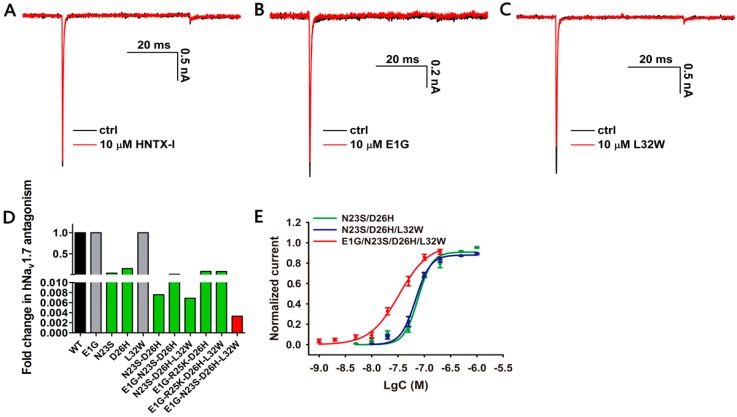
Inhibitory activity of HNTX-I analogues on Na_V_1.7. (**A**) Representative Na_V_1.7 current trace before (black) and after (red) addition of 10 µM wild-type HNTX-I; (**B**) Representative Na_V_1.7 current trace before (black) and after (red) addition of 10 µM E1G; (**C**) Representative Na_V_1.7 current trace before (black) and after (red) addition of 10 µM L32W; (**D**) Fold change of inhibitory effect of wild-type HNTX-I and HNTX-I analogues on Na_V_1.7; (**E**) Concentration–response curves of N23S–D26H, N23S–D26H–L32W and E1G–N23S–D26H–L32W analogues assessed by whole-cell patch clamp. Data are mean ± SEM, with *n* = 3–5 cells per data point.

As mentioned above, we conducted amino acid substitutions at the following positions in HNTX-I. Asparagine in position 23 was mutated to serine (N23S), and aspartic acid in position 26 was mutated to histidine (D26H). In the NaSpTx family 1, a triple mutant of HWTX-IV (E1G, E4G, Y33W–HWTX–IV) showed a great increase of potency against Na_V_1.7 [24]. Therefore, we mutated glutamic acid in position 1 to glycine (E1G) and leucine in position 32 to tryptophan (L32W) in the hopes of increasing affinity to Na_V_1.7. However, similar to wild-type HNTX-I, E1G and L32W had negligible activity on Na_V_1.7 at a concentration of 10 µM (Figure 2A–C). Notably, N23S showed an obvious increase in activity (IC_50_ 0.435 ± 0.072 µM), tenfold higher than previously reported [22]. In addition, D26H conferred Na_V_1.7-inhibitory activity on HNTX-I with an IC_50_ value of 1.498 ± 0.093 µM. Therefore, N23S and D26H individually contributed to inhibition of hNa_V_1.7 (Figure 2D and Table 1).

It has been reported that the activity of a combination of mutants is not necessarily better than that of a single mutant [25]. We combined the single mutants described above to see whether activity would increase significantly. As shown in Figure 2D,E and Table 1, N23S–D26H demonstrated an obvious increase in potency (IC_50_ 0.079 ± 0.004 µM) against hNa_V_1.7, compared with individual mutants. However, the combination of N23S–D26H with E1G (E1G–N23S–D26H) reduced activity (IC_50_ 0.179 ± 0.024 μM), while the mutant into which N23S–D26H and L32W were combined (N23S–D26H–L32W) exhibited activity (IC_50_ 0.071 ± 0.005 µM) close to that of N23S–D26H. We also tried to combine these single mutants together. Ultimately, the mutant E1G–N23S–D26H–L32W caused the greatest increase in activity (IC_50_ 0.036 ± 0.007 µM). The dose–response curves of N23S–D26H, N23S–D26H–L32W and E1G–N23S–D26H–L32W are shown in Figure 2E.

Meanwhile, we also tried to replace arginine in position 25 with the more popular lysine at the same charge. Neither E1G–R25K–D26H (IC_50_ 0.766 ± 0.029 µM) nor E1G–R25K–D26H–L32W (IC_50_ 0.669 ± 0.070 µM) showed stronger activity than did E1G–N23S–D26H–L32W (Figure 2D and Table 1). In summary, we successfully engineered a gain-of-function analogue of HNTX-I with potent inhibitory effect on mammalian sodium channel hNa_V_1.7 by the method of sequence conservation in the family.

### 2.3. Structure–Activity Relationship of HNTX-I and Mutant

**Figure 3:**
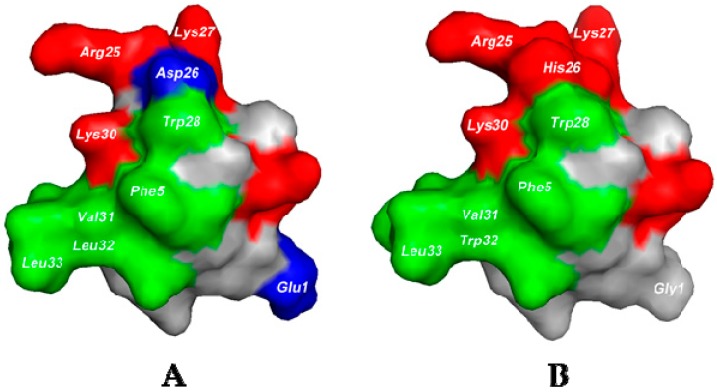
Surface rendering of NMR structure of HNTX-I and HNTX-I analogue E1G–N23S–D26H–L32W. (**A**) Surface rendering of HNTX-I (PDB code 1NIX); (**B**) Surface rendering of a homology model of HNTX-I analogue E1G–N23S–D26H–L32W. Positively charged residues are shown in red, negatively charged residues in blue, hydrophobic residues in green, and polar uncharged residues in gray. This figure was generated using PyMOL.

We have added the NMR solution structure of HNTX-I to the Protein Data Bank (Figure 3A; PDB code 1NIX; www.rcbs.org/pdb). In HNTX-I’s structure, the basic residues Arg25, Lys27 and Lys30 clustered to form a positively charged surface and interacted with VGSCs by electrostatic interaction. The vicinal hydrophobic residues Phe5, Tyr20, Trp28, Val31, Leu32 and Leu33 clustered to form a hydrophobic patch. Both the hydrophobic and the positively charged residues are highly conserved in NaSpTx family 1. We modeled the 3-D structure of E1G–N23S–D26H–L32W using that of HNTX-I as a template (Figure 3B). After we mutated acidic residue Asp26 to basic residue His26, the intensity of the electrostatic interaction of the original positively charged surface was enhanced. In addition, Gly1 reduced the negative charge. All these factors may affect the electrostatic properties of the peptide and change its pharmacological specificity. Furthermore, the aromatic group Trp32 strengthened hydrophobic interaction in the hydrophobic patch, and S23 was required to orient the critical Trp29 and Lys31 residues correctly [20,26]. Taken together, these results suggest that in addition to the highly conserved charged surface and the hydrophobic surface, less-conserved acidic residues and surrounding residues play important roles in pharmacological flexibility.

## 3. Discussion

The VGSC Na_V_1.7 is preferentially expressed in peripheral sensory neurons. Genetic, preclinical animal models and functional studies have shown that Na_V_1.7 regulates sensory-neuron excitability and is a major contributor to several sensory modalities [27]. Because of the key role of Na_V_1.7 in the nervous system, it is a hot target for the action of peptide toxins derived from defensive or predatory animal venoms such as HWTX-IV, HNTX-IV and µ-SLPTX-Ssm6a. These toxins have exhibited remarkable analgesia in several animal models [28,29,30]. Therefore, the hNa_V_1.7 channel is considered an analgesic target and is the focus of drug design. Peptide toxins inhibiting Na_V_1.7 activation are remarkably similar; they trap the voltage sensor of domain II s3b-s4, which is a hot-paddle motif in Na_V_1.7 [31,32]. The mutations in s3b-s4 significantly disrupt the binding of toxins, especially charged amino acids D816 and E818 located in domain II s3b-s4 [32,33]. This evidence suggests that peptide toxins inhibit Na_V_1.7 by use of conventional approaches and that similar activity domains may exist on peptide toxins.

In this study, we engineered HNTX-I and explored its analogues’ inhibitory activity against established pain target hNa_V_1.7. Recently, the X_1_X_2_SWCKX_3_ motif, in which X stands for any hydrophobic residues and S is required to position W and K correctly, was proposed to be essential for the activity of peptides on Na_V_1.7 [26]. In our study, the gain-of-function analogue E1G–N23S–D26H–L32W achieved high potency against Na_V_1.7 with an IC_50_ value of 0.036 ± 0.007 µM, and its structure pattern conformed to the proposed motif. E1G–N23S–D26H–L32W also exhibited inhibitory activity on Na_v_1.2 and Na_v_1.6 channels (Figure S3), and further studies are required to optimize Na_v_ subtype selectivity for potential therapeutic use. Residue in position 26 was not conserved as well as it was in positions 1 and 32. These variable residues may determine pharmacological specificity against insect or mammalian VGSCs. His26 clustering with Arg25, Lys27 and Lys30 formed the charged surface, and Trp32 together with surrounding residues Phe5, Tyr20, Trp28, Val31 and Leu33 combined to form the hydrophobic surface. These surfaces together determined and modified the peptide’s binding affinity to the Na_V_1.7 channel. Moreover, these key residues together with the X_1_X_2_SWCKX_3_ motif can be used as an important scaffold when designing or modifying peptides with structure patterns similar to that of the NaSpTx family 1 in the future. Engineering peptides based on sequence and structure conservation in the family appears to be a more economical and efficient way than positional scanning with representative members of different classes of amino acids. The strategy of combining structure–activity relationships and the conservation of sequence evolution will together provide better insight into peptide drug design.

## 4. Materials and Methods

### 4.1. Peptide Synthesis, Oxidative Folding, Purification and Characterization

We synthesized HNTX-I analogues using a fluorenylmethyloxycarbonyl protecting group (Fmoc; *N*-(9-Fluorenyl)methoxycarbonyl)/tert-butyl strategy and a coupling method involving 1-hydroxy-benzotriazole (HOBt), *O*-(benzotriazol-1-yl)-1,13,3-tetramethyluronium hexafluorophosphate (TBTU) and *N*-methylmorpholine (NMM) [34]. We then purified the linear peptides by semi-preparative reverse-phase high-performance liquid-chromatography (RP-HPLC) purification (C_18_ column, 10 mm × 250 mm; Welch Materials Inc., Shanghai, China) with a 20-min linear acetonitrile gradient of 20–40% at a 3 mL/min flow rate (Hanbon HPLC system equipped with NP7000 serials pump and NU3000 serials UV/VIS detector, Hanbon Science & Technology Co., Ltd., Huai’an, China). The refolding buffer contained (in mM): 100 NaCl, 5 glutathione (GSH), 0.5 glutathione disulfide (GSSG) and 100 Tris (pH = 8.0, adjusted with NaOH). We diluted the purified linear peptide with the refolding buffer to a final concentration of 0.1 mg/mL, slowly stirred the solution at 25 °C for 24 h, and monitored the refolding reaction by matrix-assisted laser desorption/ionization (MALDI)–time–of–flight (TOF) mass spectrometry (MS) analysis (AB SCIEX TOF/TOF^TM^ 5800 system; Applied Biosystems, Foster City, CA, USA). We terminated the reaction by adding trifluoroacetic acid (TFA) to a final concentration of 0.2% and isolated the desired oxidized peptide by RP-HPLC purification (C_18_ column, 4.6 mm × 250 mm; Welch Materials, Inc., Shanghai, China) using a 20-min linear acetonitrile gradient of 20–40% at a 1 mL/min flow rate (Hanbon HPLC system equipped with NP7000 serials pump and NU3000 serials UV/VIS detector; Hanbon Science & Technology Co., Ltd., Huai’an, China). The synthesis and oxidative folding of wild-type HNTX-I and E1G-N23S-D26H-L32W were shown in Supplementary Materials.

### 4.2. Cell Culture and Transfection

We maintained human embryonic kidney (HEK) 293 cells at 37 °C in a humidified 5% CO_2_ incubator in Dulbecco’s Modified Eagle’s Medium (DMEM) supplemented with 10% fetal bovine serum (FBS), 2 mM l-glutamine, 100 units/mL penicillin and 100 µg/mL streptomycin. cDNA genes encoding rat Na_v_1.2 and mouse Na_v_1.6 were subcloned into the vectors pcDNA3.1, respectively. We subcloned the gene that encodes hNa_V_1.7 into the vector pcDNA3.1-mod [35]. We transfected the hNa_V_1.7 channel (3 μg) together with plasmids β1- (1 μg) and β2-eGFP (1 vg), which encode the human β1 and β2 subunits, and Na_v_1.2 and Na_v_1.6 together with eGFP in HEK 293 cells using Lipofectamine 2000 (Invitrogen, Carlsbad, CA, USA) per the manufacturer’s instructions.

### 4.3. Whole-Cell Patch Clamp Recordings

We performed whole-cell recordings using an EPC 10 patch clamp platform (HEKA, Elektronik, Lambrecht, Germany) at room temperature (20–25 °C). We made suction pipettes with access resistance of 2.0–3.0 MΩ from borosilicate glass capillary tubes (VWR micropipettes; VWR Co., West Chester, PA, USA) using a 2-step vertical microelectrode puller (PC-10; Narishige Co., Ltd., Tokyo, Japan) and compensated for voltage errors using 80% serial-resistance compensation. The standard pipette solution contained (in mM): 140 CsCl, 10 NaCl, 1 ethylene glycol-bis (β-aminoethyl ether)-*N*,*N*,*N*′,*N*′-tetraacetic acid (EGTA), and 10 HEPES (pH 7.4). The bath solution contained (in mM): 140 NaCl, 2 CaCl_2_, 1 MgCl_2_, 5 KCl, 20 4-(2-hydroxyethyl)-1-piperazineethanesulfonic acid (HEPES) and 10 glucose (pH 7.4). We purchased all chemicals from Sigma-Aldrich (St. Louis, MO, USA) and dissolved them in water. Data were acquired using PATCHMASTER software version 2x73 (HEKA Elektronik Dr. Schulze GmbH, Lambrecht, Germany). We filtered macroscopic sodium currents at 5 kHz and sampled them at 20 kHz. We acquired voltage clamp recordings 5 min after establishing a whole-cell configuration to allow adequate equilibration between the micropipette solution and the cell interior. We elicited channel current by 50 milliseconds of depolarization potential to −10 mV from the holding voltage of −100 mV.

### 4.4. Molecular Model of E1G-N23S-D26H-L32W

We modeled the 3-dimensional structure of E1G–N23S–D26H–L32W using the nuclear magnetic resonance (NMR)–derived structure of HNTX-I (PBD code 1NIX) as a template [36]. We performed backbone fitting and energy minimization using the Swiss Model prediction algorithm (open source; http://swissmodel.expasy.org) [37] and displayed them with PyMOL Molecular Graphics System software version 1.7.2 (Schrödinger, LLC, New York, NY, USA). The model was validated by inspection of the Ramachandran plot.

### 4.5. Data Analysis

We analyzed the data using Igor Pro software version 6.10A (WaveMetrics, Lake Oswego, OR, USA), Sigmaplot software version 10 (Sigma, St. Louis, MO, USA), OriginPro software version 8 (OriginLab Corp., Northampton, MA, USA) and Prism software version 5 (GraphPad Software, San Diego, CA, USA). Concentration–response curves were fitted using the following Hill logistic equation:

*y* = *f_max_* − (*f_max_* − *f_min_*)/(1 + (*x*/IC_50_)*^n^*) (1)

where *f_max_* and *f_min_* respectively represent the channel’s maximum and minimum responses to toxins, *f_min_* was set to 0, *x* represents toxin concentration and *n* is an empirical Hill coefficient. All quantitative results are presented as mean ± standard error of the mean (SEM).

## Figures and Tables

**Table 1:** IC_50_ value of HNTX-I analogues determined by whole-cell patch clamp *.

Peptide	Amino Acid Sequence	hNa_V_1.7 IC_50_ (µM)
Native HNTX-I	ECKGFGKSCVPGKNECCSGYACNSRDKWCKVLL	>10
E1G	GCKGFGKSCVPGKNECCSGYACNSRDKWCKVLL	>10
N23S	ECKGFGKSCVPGKNECCSGYACSSRDKWCKVLL	0.435 ± 0.072
D26H	ECKGFGKSCVPGKNECCSGYACNSRHKWCKVLL	1.498 ± 0.093
L32W	ECKGFGKSCVPGKNECCSGYACNSRDKWCKVWL	>10
N23S–D26H	ECKGFGKSCVPGKNECCSGYACSSRHKWCKVLL	0.079 ± 0.004
E1G–N23S–D26H	GCKGFGKSCVPGKNECCSGYACSSRHKWCKVLL	0.179 ± 0.024
E1G–R25K–D26H	GCKGFGKSCVPGKNECCSGYACNSKHKWCKVLL	0.766 ± 0.029
N23S–D26H–L32W	ECKGFGKSCVPGKNECCSGYACSSRHKWCKVWL	0.071 ± 0.005
E1G–R25K–D26H–L32W	GCKGFGKSCVPGKNECCSGYACNSKHKWCKVWL	0.669 ± 0.070
E1G–N23S–D26H–L32W	GCKGFGKSCVPGKNECCSGYACSSRHKWCKVWL	0.036 ± 0.007

*—Data are presented as mean ± SEM, *n* ≥ 3. Amino acid residues in red color represented the mutated residues.

## Supplementary Materials

The following are available online at http://www.mdpi.com/2072-6651/10/9/358/s1, Figure S1: Synthesis and oxidative folding of wild type HNTX-I, Figure S2: Synthesis and oxidative folding of HNTX-I analogue E1G-N23S-D26H-L32W, Figure S3: Effect of E1G-N23S-D26H-L32W on Na_v_1.2 and Na_v_1.6 expressed in HEK 293 cells.
