# Supplementary material for: Engineering Gain-of-Function Analogues of the Spider Venom Peptide HNTX-I, A Potent Blocker of the hNaV1.7 Sodium Channel
Source: Toxins (Basel). 2018 Sep 4;10(9):358. doi: 10.3390/toxins10090358 (PMC6162447; doi:10.3390/toxins10090358)
Supplement: Supplementary file 1 [file toxins-10-00358-s001.pdf]

Supplementary Materials: Engineering Gain-of-Function Analogues of the Spider Venom Peptide HNTX-I, A Potent Blocker of the hNav1.7 Sodium Channel

Yunxiao Zhang , Qiuchu Yang , Qingfeng Zhang, Dezheng Peng, Minzhi Chen, Songping Liang, Xi Zhou and Zhonghua Liu

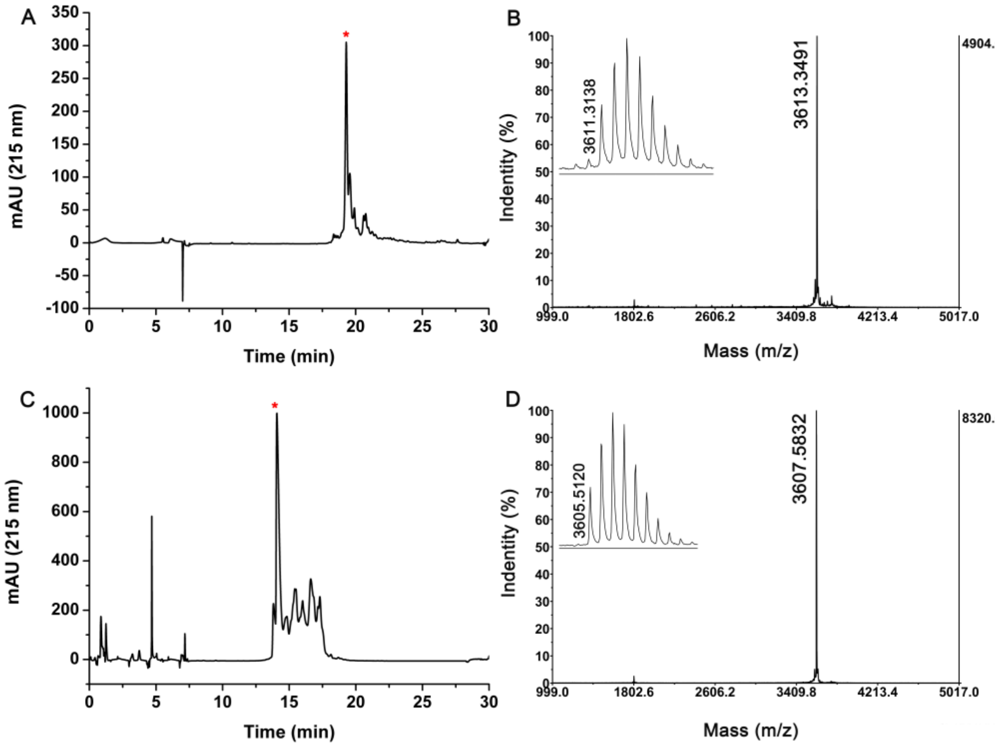

Figure S1. Synthesis and oxidative folding of wild type HNTX-I. (**A**) RP-HPLC purification of crude linear HNTX-I, and asterisk indicated the peak containing HNTX-I linear peptide; (**B**) MALDI-TOF MS analysis of purified linear HNTX-I; (**C**) Analytical RP-HPLC purification of crude folded HNTX-I, and asterisk indicated the correctly folded peptide; (**D**) MALDI-TOF MS analysis of the purified folded HNTX-I, and inset was an enlarged view of the peak.

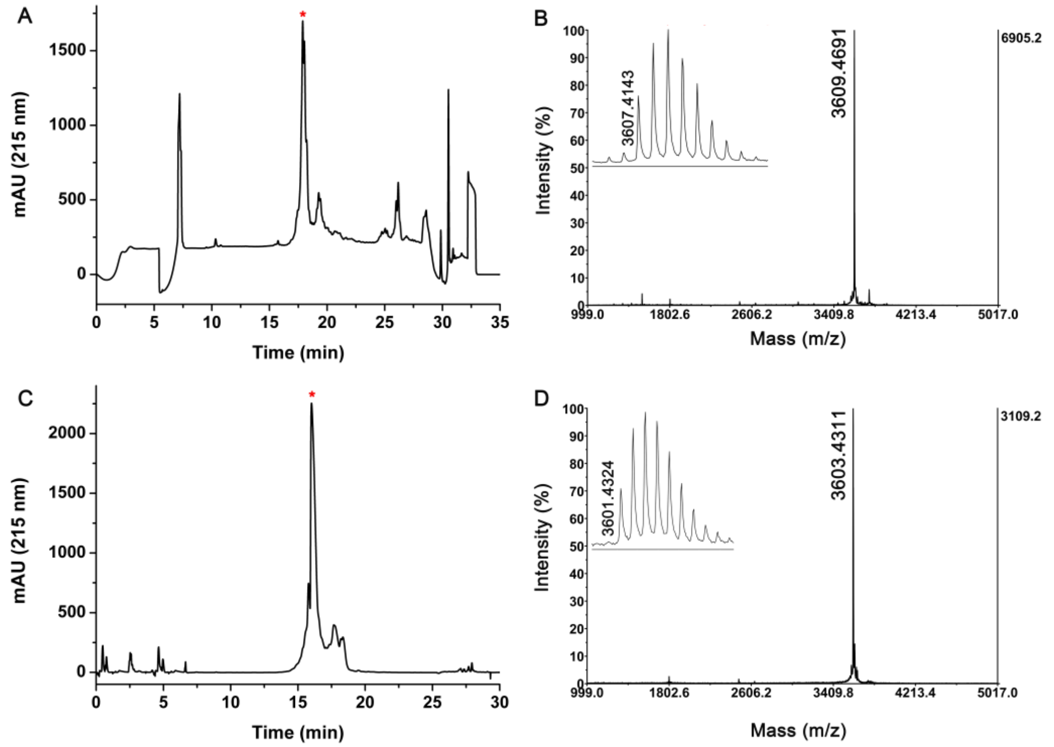

Figure S2. Synthesis and oxidative folding of HNTX-I analogue E1G-N23S-D26H-L32W. (**A**) RP-HPLC purification of crude linear HNTX-I analogue E1G-N23S-D26H-L32W, and asterisk indicated the peak containing E1G-N23S-D26H-L32W linear peptide; (**B**) MALDI-TOF MS analysis of purified linear E1G-N23S-D26H-L32W; (**C**) Analytical RP-HPLC purification of crude folded E1G-N23S-D26H-L32W, and asterisk indicated the correctly folded peptide; (**D**) MALDI-TOF MS analysis of the purified folded HNTX-I, and inset was an enlarged view of the peak.

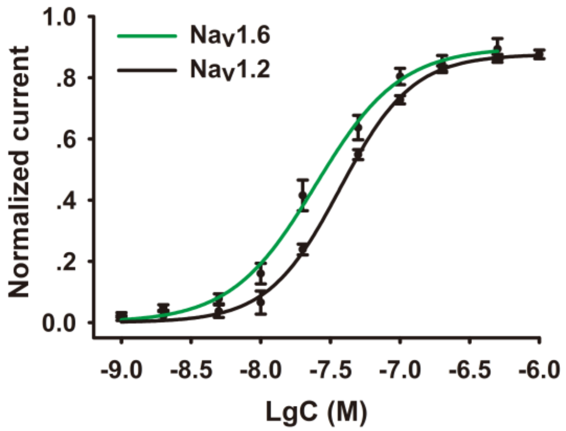

Figure S3. Effect of E1G-N23S-D26H-L32W on $Na_v1.2$ and $Na_v1.6$ expressed in HEK 293 cells. Concentration-response curves of E1G-N23S-D26H-L32W at $Na_v1.2$ and $Na_v1.6$ assessed by whole-cell patch-clamp experiments. IC_{50} value of E1G-N23S-D26H-L32W on $Na_v1.2$ and $Na_v1.6$ werev Data are mean ± SEM, with *n* = 3–5 cells per data point.
